# Salivary Galectin-9 Levels in Primary Sjögren’s Disease: An Observational Cross-Sectional Case–Control Study

**DOI:** 10.3390/jcm15103684

**Published:** 2026-05-11

**Authors:** Elif İnanç, Servet Yolbaş, Sezgin Zontul, Fuat Albayram, Mesude Seda Aydoğdu, Zeynep Kaya, Faruk Dişli, Cihat Uçar

**Affiliations:** 1 Division of Rheumatology, Department of Internal Medicine, Van Training and Research Hospital, University of Health Sciences, 65300 Van, Turkey; 2 Division of Rheumatology, Department of Internal Medicine, Faculty of Medicine, Inonu University, 44280 Malatya, Turkey; 3 Division of Rheumatology, Department of Physical Therapy and Rehabilitation, Faculty of Medicine, Inonu University, 44280 Malatya, Turkey; 4 Division of Rheumatology, Department of Internal Medicine, Malatya Training and Research Hospital, 44330 Malatya, Turkey; 5 Division of Rheumatology, Department of Internal Medicine, Istanbul Training and Research Hospital, 34098 Istanbul, Turkey; 6 Department of Physical Therapy and Rehabilitation, Faculty of Physical Therapy and Rehabilitation, Bingol University, 12000 Bingol, Turkey; 7 Department of Physiology, Faculty of Medicine, Malatya Turgut Ozal University, 44210 Malatya, Turkey

**Keywords:** primary Sjögren’s disease, galectin-9, biomarker, disease activity

## Abstract

**Background/Objectives**: Primary Sjögren’s disease is a systemic autoimmune disease characterized by chronic inflammation of exocrine glands and heterogeneous clinical manifestations. There remains a need for objective, non-invasive biomarkers that reflect local glandular involvement and disease-related immune activity. **Methods**: This observational cross-sectional case–control study included 34 patients fulfilling the 2016 ACR/EULAR classification criteria for primary Sjögren’s disease and 34 healthy controls between December 2024 and February 2025. Unstimulated whole-saliva samples were collected in the morning using the passive drool method, and salivary galectin-9 concentrations were measured via the enzyme-linked immunosorbent assay. Disease activity and symptom burden were assessed using validated indices, and receiver operating characteristic analysis was performed to evaluate discriminatory performance. **Results**: Salivary galectin-9 levels were significantly higher in patients with primary Sjögren’s disease compared with healthy controls. However, no significant associations were observed between salivary galectin-9 levels and disease activity scores after correction for multiple comparisons, nor with patient-reported symptoms, autoantibody profiles, Schirmer test results, or minor salivary gland biopsy findings. Salivary galectin-9 demonstrated limited discriminative ability between patients and controls. **Conclusions**: Salivary galectin-9 levels were elevated in primary Sjögren’s disease and may be associated with local glandular immune processes. Further prospective studies are needed to determine their clinical relevance.

## 1. Introduction

Primary Sjögren’s disease (pSD) is an autoimmune disease characterized by chronic inflammation of exocrine glands, leading to ocular and oral dryness and potential systemic involvement. Inflammation of the salivary and lacrimal glands results in glandular dysfunction, while widespread immunological activity may develop over time and affect multiple organ systems [1]. Ocular and oral dryness are the most commonly reported symptoms, and nearly all patients experience at least one of these complaints [1].

The clinical and biological spectrum of the disease is broad. While some patients present only with glandular symptoms, others may exhibit significant systemic involvement and impaired quality of life. This heterogeneity complicates both diagnosis and assessment of disease activity. Although scoring systems such as the EULAR Sjögren’s Syndrome Disease Activity Index (ESSDAI) and EULAR Sjögren’s Syndrome Patient-Reported Index (ESSPRI) are routinely used in clinical practice, they do not always fully reflect real-time immunological activity [2,3].

Therefore, there remains a need for objective, rapid, accessible, and non-invasive biomarkers. In this context, saliva is not merely a glandular secretion but also a complex biological fluid containing various blood-derived molecules, including enzymes, hormones, antibodies, and inflammatory mediators. Thus, saliva has the potential to reflect systemic physiological changes, local exocrine inflammation, and glandular immune responses. This is particularly relevant in pSD, as the disease primarily targets the salivary glands and is characterized by local lymphocytic infiltration and glandular inflammation. While serum biomarkers mainly reflect systemic immune activity, saliva may more directly reflect glandular secretions and local inflammatory processes. Therefore, salivary biomarkers may provide complementary and more specific information regarding local glandular involvement compared with serum-based measurements. In addition, saliva sampling is non-invasive, easily repeatable, and practical for clinical use. For these reasons, saliva represents a biologically meaningful and feasible sample for evaluating local glandular involvement in pSD [4].

Galectin-9 (Gal-9) is a β-galactoside-binding lectin that serves as an important regulator of innate and adaptive immunity. It is expressed in various immune cells—including T cells, B cells, and dendritic cells—and plays critical roles in immune tolerance, inflammation, and apoptosis [5,6].

The pathogenesis of pSD has not been fully elucidated. Increased Gal-9 levels have been reported in several autoimmune diseases, including systemic lupus erythematosus (SLE), systemic sclerosis, rheumatoid arthritis (RA), juvenile dermatomyositis, and pSD, and Gal-9 has been shown to be strongly associated with the interferon signature [5,7]. These findings suggest that Gal-9 may be linked to interferon-mediated inflammatory mechanisms and contribute to the pathogenesis of pSD. Moreover, serum Gal-9 has been suggested as a potential marker for predicting treatment response [8].

Most existing studies have focused on serum Gal-9 levels, and data on salivary Gal-9 remain limited. However, saliva may reflect early stages of glandular inflammation. Therefore, evaluating Gal-9 in saliva could represent a novel approach for early diagnosis, activity monitoring, and prediction of treatment response in pSD.

In this study, we aimed to measure salivary Gal-9 levels in patients with pSD, compare them with healthy controls, and evaluate their relationship with disease activity.

## 2. Materials and Methods

### 2.1. Study Population

This cross-sectional study included 34 patients with primary Sjögren’s disease (pSD) who presented to the rheumatology outpatient clinic between December 2024 and February 2025 and fulfilled the 2016 EULAR/ACR classification criteria, as well as 34 healthy controls. Healthy controls were individuals attending routine outpatient evaluation without sicca symptoms or a history of rheumatologic or systemic autoimmune diseases.

The study protocol was approved by the Clinical Research Ethics Committee of İnönü University (11 December 2024; Decision No: 2024/164) and was conducted in accordance with the principles of the Declaration of Helsinki.

Individuals younger than 18 years or older than 65 years, those with active infection, malignancy, or other rheumatologic diseases, and those using medications known to affect salivary secretion (including antihistamines, antidepressants, or beta-blockers) were excluded from this study.

### 2.2. Clinical and Laboratory Assessment

Demographic characteristics, clinical features, laboratory parameters, disease activity scores, medication use, and organ involvement patterns were recorded for patients. During the clinical assessment, self-reported history of periodontal disease was also recorded. Demographic and laboratory data were similarly collected for the control group. Laboratory assessments included complete blood count, renal function tests, C-reactive protein (CRP), erythrocyte sedimentation rate (ESR), rheumatoid factor (RF), antinuclear antibodies (ANA), anti-SSA, anti-SSB, and complement C3 and C4 levels. Schirmer test results and minor salivary gland biopsy findings were obtained from medical records when available.

Disease activity was assessed using the EULAR Sjögren’s Syndrome Disease Activity Index (ESSDAI). Patients with an ESSDAI score of 0 were considered to have inactive disease, whereas those with an ESSDAI score ≥ 1 were classified as having active disease. Disease activity was further categorized as low (ESSDAI < 5), moderate (5 ≤ ESSDAI ≤ 13), or high (ESSDAI ≥ 14) [9]. Patient-reported symptom burden was evaluated using the EULAR Sjögren’s Syndrome Patient-Reported Index (ESSPRI), with a score ≥ 5 indicating an unacceptable symptom burden [3].

### 2.3. Saliva Collection and Laboratory Analyses

Unstimulated whole-saliva samples were collected between 08:00 and 10:00 a.m. using the passive drool method into sterile 1.5 mL polypropylene tubes over approximately 15 min. All participants were informed about the sampling protocol and instructed to refrain from eating or drinking (except water), brushing their teeth, or smoking for at least one hour prior to sample collection.

Collected saliva samples were immediately stored at −80 °C until analysis. On the day of analysis, samples were thawed at room temperature and centrifuged at 4000× *g* for 10 min, and the supernatant was used for subsequent enzyme-linked immunosorbent assay (ELISA) measurements. According to the assay protocol, samples were diluted 1:100 prior to analysis. This dilution factor was selected in accordance with the manufacturer’s protocol and ensured that the measured salivary Gal-9 concentrations fell within the working range of the standard curve.

Salivary Gal-9 concentrations were measured using a commercially available ELISA kit (Human GAL-9 ELISA Kit, Elabscience; Cat. No: E-EL-H1059; Lot No: WW03448H7445; Wuhan, China) according to the manufacturer’s instructions. According to the manufacturer’s specifications, the assay had a measurement range of 7.81–500 pg/mL and a detection limit of 4.69 pg/mL. The intra- and inter-assay coefficients of variation were below 10%. Salivary Gal-9 concentrations were evaluated as absolute concentrations, and no normalization to total protein concentration was performed. All analyses were performed in the Immunoassay Development Laboratory of the Department of Physiology, Faculty of Medicine, İnönü University.

### 2.4. Statistical Analysis

Categorical variables were expressed as frequencies and percentages, whereas continuous variables were presented as the mean ± standard deviation or median (minimum–maximum), depending on data distribution. The normality of continuous variables was assessed using the Shapiro–Wilk test. Group comparisons were performed using the independent samples *t*-test or the Mann–Whitney U test, as appropriate, and categorical variables were compared using the chi-square test.

Correlations were evaluated using Spearman’s rank correlation coefficient. A two-tailed *p* value < 0.05 was considered statistically significant for primary analyses. For multiple correlation analyses involving salivary Gal-9, statistical significance was determined based on Benjamini–Hochberg false discovery rate (FDR)-adjusted *p* values (*q* < 0.05). The discriminatory performance of salivary Gal-9 was assessed using receiver operating characteristic (ROC) analysis, with the optimal cut-off value determined using the Youden *J* statistic. Area under the curve (AUC) values were reported with 95% confidence intervals and compared using the DeLong test. Age-adjusted binary logistic regression analysis was performed to assess the independent association between salivary Gal-9 levels and primary Sjögren’s disease. Sample size calculation was performed using G*Power version 3.1, assuming an alpha level of 0.05, a statistical power of 0.80, an effect size of 0.70, and a 1:1 allocation ratio, yielding a required sample size of 34 participants per group. All statistical analyses were conducted using IBM SPSS Statistics version 26.0 (IBM Corp., Armonk, NY, USA).

## 3. Results

### 3.1. Demographic Characteristics of Participants

The demographic and laboratory characteristics of the pSD patients and healthy controls included in the study are presented in Table 1. A significant difference was observed in terms of age, with the mean age of the pSD group being significantly higher than that of the control group (49.53 ± 9.33 vs. 41.76 ± 9.61 years; *p* < 0.001). Sex distribution was similar between the groups; the proportion of females was 94.1% in the pSD group and 91.2% in the control group (*p* = 0.64).

There was no significant difference between the groups regarding body mass index (BMI) (*p* = 0.07). Likewise, the frequency of smoking did not differ significantly between the groups (*p* = 0.15). However, the presence of comorbidities was significantly higher in the pSD group compared with the control group (58.8% vs. 14.7%; *p* < 0.001) (Table 1). Self-reported periodontal disease history was present in 5/34 (14.7%) patients with pSD and 4/34 (11.8%) healthy controls. In the overall study population, salivary Gal-9 levels did not differ significantly according to self-reported periodontal disease history (*p* = 0.101). No significant correlation was observed between age and salivary Gal-9 levels in either the pSD group (Spearman ρ = 0.148, *p* = 0.402) or the control group (Spearman ρ = 0.070, *p* = 0.696). In addition, in age-adjusted binary logistic regression analysis, salivary Gal-9 levels remained significantly associated with pSD status (*p* = 0.026).

### 3.2. Clinical Characteristics of Patients with Primary Sjögren’s Disease

The mean disease duration was 4.15 ± 4.39 years. All patients had mucosal involvement (100%), and articular involvement was the most common systemic manifestation (88.2%). Other organ involvements—including cutaneous, lymph node, hepatic, hematologic, parotid, and pulmonary involvement—were rare (each < 6%).

Immunological evaluation revealed antinuclear antibody (ANA) positivity in 82.4% of patients, anti-SSA positivity in 55.9% (19/34), anti-SSB positivity in 29.4%, and rheumatoid factor positivity in 32.4%, while low complement levels were observed in only two patients (5.9%). Minor salivary gland biopsy was performed in 19 patients, of whom 17 showed positive histopathological findings with a focus score ≥ 1 (17/19, 89.5%; overall 17/34, 50.0%). In patients who underwent biopsy, the distribution of focus scores was as follows: focus score 0 in 2 patients (10.5%), focus score 1 in 12 patients (63.2%), focus score 2 in 3 patients (15.8%), and focus score 3 in 2 patients (10.5%). No significant correlation was found between salivary Gal-9 levels and focus score (Spearman’s rho = −0.068, *p* = 0.701). Schirmer test positivity was observed in 85.3% of patients (Table 2).

The most frequently used treatments were glucocorticoids (55.9%) and hydroxychloroquine (47.1%). Among immunosuppressive agents, azathioprine was used by 26.5% of patients, leflunomide by 11.8%, methotrexate by 8.8%, and tacrolimus or mycophenolate mofetil by 2.9% each. Pilocarpine was administered in 11.8% of patients, and none had a history of biological therapy use.

With respect to disease activity, the mean ESSDAI score was 1.88 ± 0.95, and disease activity defined as an ESSDAI score ≥ 1 was present in 61.8% of the patients. Within this group, low disease activity (ESSDAI < 5) was observed in 50.0% and moderate disease activity (5 ≤ ESSDAI ≤ 13) in 11.8%, while no patients exhibited high disease activity. The mean ESSPRI score was 6.13 ± 1.91 (range: 1–10), and the majority of participants had a clinically unacceptable symptom burden (ESSPRI ≥ 5). The patient global assessment score was 5.82 ± 2.02, and the physician global assessment score was 4.29 ± 1.12 (Table 2).

### 3.3. Laboratory Findings

Examination of laboratory parameters revealed that the erythrocyte sedimentation rate (ESR) was significantly higher in the pSD group compared with the control group (11.15 ± 8.27 mm/h vs. 7.00 ± 5.18 mm/h; *p* = 0.03). Neutrophil count was significantly lower in patients with pSD compared with controls (3.51 [2.64–4.24] × 10^9^/L vs. 4.15 [3.91–5.11] × 10^9^/L; *p* < 0.01). No significant differences were observed between the groups in other hematological parameters—including leukocyte, lymphocyte, monocyte, platelet counts, hemoglobin, and creatinine levels (all *p* > 0.05). One of the most notable findings of this study was that salivary Gal-9 levels were significantly higher in patients with pSD compared with healthy controls. Given the skewed distribution of the data, median values were considered, revealing Gal-9 levels of 145.9 pg/mL (range: 0–1050) in the pSD group and 63.3 pg/mL (range: 0–381.2) in the control group (*p* < 0.001).

### 3.4. Subgroup Analyses in Patients with Primary Sjögren’s Disease

In the subgroup analyses, Gal-9 levels were evaluated according to disease activity (ESSDAI present/absent), medication use (steroids, hydroxychloroquine, azathioprine, methotrexate, leflunomide, tacrolimus, mycophenolate mofetil, pilocarpine), clinical involvement domains (articular, cutaneous, parotid, hematologic/visceral), autoantibody positivity (ANA, SSA, SSB, RF), complement levels, Schirmer test results, and minor salivary gland biopsy findings. Salivary Gal-9 levels were not significantly different between patients with ESSDAI-defined disease activity (ESSDAI ≥ 1) and those without disease activity (*p* = 0.071). In subgroup analyses based on ESSDAI activity levels (low and moderate activity), no significant differences in Gal-9 levels were observed (*p* = 0.370). No significant differences in Gal-9 levels were observed with respect to clinical involvement domains (articular, cutaneous, parotid, hematologic, or visceral), autoantibody positivity (ANA, SSA, SSB, RF), low complement levels, biopsy or Schirmer test results, or the use of immunosuppressive medications (all *p* > 0.05).

In the pSD group, no statistically significant association was observed between salivary Gal-9 levels and the ESSDAI disease activity score after Benjamini–Hochberg false discovery rate correction (FDR-adjusted *p* = 0.669). Before correction, a nominal weak negative correlation was observed (ρ = −0.342; *p* = 0.048) (Table 3). This finding was therefore considered exploratory in nature. No significant association was found between Gal-9 levels and the ESSPRI patient-reported symptom score. Age was not significantly associated with salivary Gal-9 levels. Similarly, no significant correlations were observed between Gal-9 levels and routine hematological or biochemical parameters, including erythrocyte sedimentation rate, C-reactive protein, leukocyte, neutrophil, monocyte, platelet, lymphocyte counts, or hemoglobin levels (Table 3).

### 3.5. ROC Analysis

**Figure 1:**
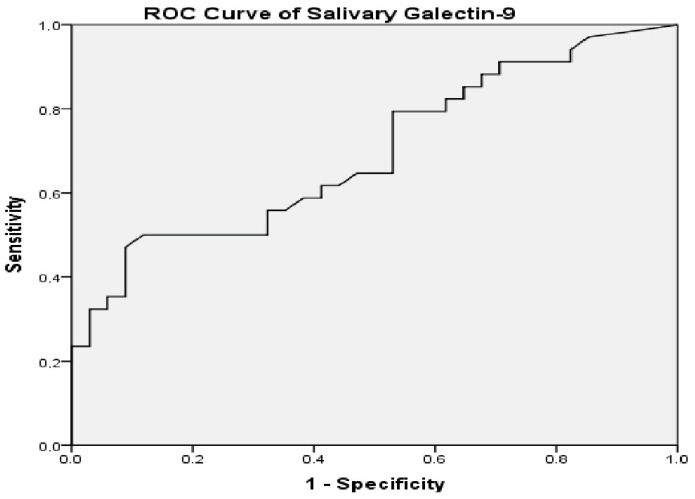
Receiver operating characteristic (ROC) curve of salivary galectin-9 levels in primary Sjögren’s disease. AUC = 0.695 (95% CI: 0.570–0.820, *p* = 0.006). The optimal cut-off value was 22.1 pg/mL, with 91.2% sensitivity and 26.5% specificity.

The ability of salivary Gal-9 levels to discriminate between the pSD and control groups was evaluated using receiver operating characteristic (ROC) analysis (pSD: n = 34; controls: n = 34). The analysis revealed an area under the curve (AUC) of 0.695 (95% CI: 0.570–0.820; DeLong test *p* = 0.006), indicating a modest discriminative performance. According to the Youden J statistic, the optimal cut-off value was 22.1 pg/mL, yielding a sensitivity of 91.2% and a specificity of 26.5% at this threshold (Figure 1).

## 4. Discussion

In this study, salivary Gal-9 levels were evaluated in patients with pSD and were found to be significantly higher compared with healthy controls (*p* < 0.001). No statistically significant association was observed between Gal-9 levels and the ESSDAI disease activity score. Receiver operating characteristic analysis demonstrated limited discriminatory performance of salivary Gal-9 (AUC = 0.695). Additionally, neutrophil count was observed to be lower in the pSD group, which may be related to immune dysregulation and alterations in inflammatory cell dynamics associated with the disease.

In recent years, the limitations of conventional serological markers—particularly in seronegative and early-stage pSD—have increased interest in saliva-based biomarkers. Previous studies have shown elevated levels of lactate, alanine, taurine, NGAL, β2-microglobulin, annexin-A2, and several microRNAs (including let-7i-5p and miR-17-5p) in saliva [10]. These findings support the notion that saliva is an important biological medium capable of reflecting immunometabolic alterations.

Interferon (IFN)-mediated immune activation plays a central role in the pathogenesis of pSD; type I IFN predominantly affects peripheral blood, whereas type II IFN is more prominent in glandular tissue [11,12]. Gal-9 is an IFN-inducible molecule. Båve et al. demonstrated marked IFN-α-positive infiltrates in labial salivary gland biopsies from patients with pSD, suggesting that local IFN activity may be stronger than systemic IFN activation [13].

Studies conducted in autoimmune diseases other than Sjögren’s disease also support Gal-9 as a sensitive indicator of interferon-mediated inflammation. In systemic lupus erythematosus (SLE), serum and urinary Gal-9 levels have been reported to be elevated and to decrease with treatment [14], and another study demonstrated that high Gal-9 levels in newly diagnosed SLE patients were particularly associated with renal and neuropsychiatric involvement [15]. Gal-9 has also been shown to exhibit a strong correlation with the IFN signature in both SLE and antiphospholipid syndrome [16]. In systemic sclerosis, interferonogenic autoantibodies have been shown to induce prominent type I IFN activation, which is associated with clinical phenotypes [17]. In rheumatoid arthritis (RA), Gal-9 levels have been reported to be associated with disease activity, inflammation, and radiographic progression, and another RA study showed that Gal-9 may enhance cytokine production from synovial fibroblasts, suggesting a potential therapeutic target [18,19]. These data indicate that Gal-9 reflects both systemic and local immune responses across various autoimmune diseases and biologically support the findings of our study.

Studies investigating the role of Gal-9 in the pathogenesis of pSD are limited. Van den Hoogen et al. reported that serum Gal-9 levels were significantly elevated in patients with pSD and showed positive correlations with serum IgG levels and ESSDAI scores [20]. Hamkour et al. demonstrated that, in patients with active pSD, the combination of leflunomide and hydroxychloroquine reduced type I IFN-related proteins (MxA, CXCL10, and Gal-9), and that an early decrease in serum Gal-9 was strongly associated with clinical response [8]. Both studies support the association between Gal-9, systemic interferon responses, and disease activity.

Only one study has investigated salivary Gal-9 in pSD, reported as a conference abstract. In this Brazilian study including 42 patients with pSD, salivary Gal-9 levels were higher and serum levels were lower compared with healthy controls, with no significant correlation between serum and salivary Gal-9 levels. The authors also reported higher salivary Gal-9 levels in patients with anti-SSB positivity and extraglandular involvement [21].

The positive correlation reported between serum Gal-9 and the ESSDAI by van den Hoogen et al. appears to differ from our findings, in which no significant association was observed between salivary Gal-9 levels and the ESSDAI after multiple-testing correction. One possible explanation for this discrepancy may be the difference in the biological compartment being measured; while serum Gal-9 may more closely reflect systemic inflammatory burden, salivary Gal-9 may be more influenced by local glandular immune activity. In addition, unlike the study by Hamkour et al., which was conducted in patients with active pSD (mean ESSDAI ≈ 10), our cohort consisted predominantly of patients with low systemic disease activity (mean ESSDAI = 1.9), and no patients with high disease activity were included. Since ESSDAI scores were generally low in our cohort, this may have limited the ability to detect potential associations with systemic disease activity. Therefore, the absence of a significant association between salivary Gal-9 and the ESSDAI in our cohort may be related to the generally low disease activity observed in most patients. However, the absence of a correlation between salivary Gal-9 and the ESSDAI or minor salivary gland biopsy (MSGB) focus score should not be interpreted as evidence of limited biological relevance. Gal-9 is an IFN-inducible molecule and may therefore reflect IFN-related immune activation rather than structural glandular damage or overall clinical disease burden. Previous studies have shown that IFN system activation plays a central role in the pathogenesis of pSD and support the involvement of Gal-9 in IFN-related immune pathways [8]. Therefore, the lack of correlation observed in our study may suggest that salivary Gal-9 reflects a distinct immunopathological process, possibly related to IFN-mediated glandular immune activation rather than directly mirroring anatomical glandular destruction.

A strength of the study by Toche dos Santos et al. is the evaluation of Gal-9 levels in saliva, serum, and salivary gland tissue. However, the absence of increased serum Gal-9 levels and the lack of a significant association with disease activity—consistent with our findings—suggest that Gal-9 may predominantly reflect local glandular immune responses. Nevertheless, because this study was published only as a conference abstract, methodological details are limited, and the robustness of the findings cannot be fully evaluated [21].

Our study is among the few investigations evaluating salivary Gal-9 levels in pSD. The use of salivary samples provides a non-invasive, reproducible, and clinically applicable approach for assessing immune mediators. We comprehensively evaluated clinical and serological characteristics and analyzed the relationship between Gal-9 levels and disease activity indicators. Our findings contribute to the growing interest in saliva-based approaches for assessing early glandular inflammatory processes in pSD. According to ROC analysis, salivary Gal-9 demonstrated limited discriminative performance (AUC = 0.695). While the high sensitivity (91.2%) may be advantageous for non-invasive screening approaches, the low specificity (26.5%) suggests that salivary Gal-9 should be interpreted cautiously as an exploratory indicator of local glandular immune activity rather than a diagnostic biomarker.

In pSD, clinical manifestations typically begin with glandular involvement, whereas systemic features may develop later in the disease course. Therefore, serum biomarkers alone may not adequately reflect early localized disease. Saliva contains molecules directly released from target tissues and represents a suitable medium for evaluating glandular immune alterations.

This study has several limitations. Differences in age and comorbidity distributions between groups may represent potential confounding factors. However, the association between salivary Gal-9 levels and pSD remained significant after age adjustment. The absence of a non-Sjögren’s sicca group limits the evaluation of salivary Gal-9 in terms of differential diagnostic performance. Another limitation is that the study cohort consisted predominantly of patients with low-to-moderate systemic disease activity, with no patients showing high ESSDAI scores, which may have limited the ability to detect associations between salivary Gal-9 and systemic disease activity and reduced the generalizability of the findings to patients with higher disease activity. Widespread use of immunosuppressive therapies may also have influenced Gal-9 levels. Periodontal disease was assessed only by self-reported history, and no formal dental or periodontal examination was performed. Therefore, the possible effect of oral inflammatory conditions on salivary Gal-9 levels cannot be completely excluded. Salivary Gal-9 concentrations were not normalized to total protein content, as total protein concentration was not assessed. Therefore, variability in salivary flow rate and hydration status may have influenced the results. In addition, the lack of routine minor salivary gland biopsy in all patients restricted the evaluation of biopsy–Gal-9 associations. Germinal center (GC)-like structures were not systematically recorded; therefore, the relationship between Gal-9 and this histopathological feature could not be evaluated. Future studies including these structures may help to better define the prognostic and immunopathological relevance of Gal-9. The low specificity observed in the ROC analysis substantially limits the clinical applicability of salivary Gal-9 as a standalone discriminative biomarker. Additionally, the relatively small sample size may have reduced the statistical power of subgroup analyses, particularly for less frequent clinical or serological features, and may have limited the detection of more subtle differences. Although the study met the required sample size for the primary comparison, the sample size may have been insufficient for certain subgroup analyses, and non-significant findings in these analyses should be interpreted with caution. Finally, the cross-sectional design precludes causal inference. Larger, multicenter, prospective studies with age- and comorbidity-matched groups and treatment-naïve patients are required to validate these findings.

In conclusion, salivary Gal-9 levels were significantly elevated in patients with pSD. These findings suggest that salivary Gal-9 may serve as an adjunctive and exploratory marker of local glandular inflammation. Further prospective studies incorporating serial measurements and comparative serum–saliva analyses are warranted to clarify its clinical relevance.

## Figures and Tables

**Table 1:** Demographic and laboratory characteristics of the study groups.

Variable	Primary Sjögren’s Disease (*n* = 34)	Control (*n* = 34)	*p*
Age (years, mean ± SD)	49.53 ± 9.33	41.76 ± 9.61	<0.001 ^1^
BMI (kg/m^2^, mean ± SD)	27.75 ± 5.19	25.73 ± 4.63	0.071 ^1^
Sex, M/F [n (%)]	2 (5.9)/32 (94.1)	3 (8.8)/31 (91.2)	0.642 ^2^
Smoking [n (%)]	5 (14.7)	10 (29.4)	0.152 ^2^
Comorbidity [n (%)]	20 (58.8)	5 (14.7)	<0.001 ^2^
ESR (mm/h, mean ± SD)	11.15 ± 8.27	7.00 ± 5.18	0.031 ^1^
CRP (mg/dL, mean ± SD)	0.48 ± 0.26	0.41 ± 0.26	0.351 ^1^
Hemoglobin (g/dL, mean ± SD)	12.95 ± 1.54	13.62 ± 1.42	0.091 ^1^
WBC (×10^9^/L, mean ± SD)	6.63 ± 2.36	7.62 ± 2.06	0.081 ^1^
Neutrophil (×10^9^/L, median [IQR])	3.51 [2.64–4.24]	4.15 [3.91–5.11]	<0.01 ^3^
Platelets (×10^9^/L, mean ± SD)	253.2 ± 51.1	255.1 ± 54.4	0.871 ^1^
Creatinine (mg/dL, mean ± SD)	0.86 ± 0.21	0.85 ± 0.15	0.791 ^1^
Salivary Galectin-9 (pg/mL, median [min–max])	145.9 [0–1050]	63.3 [0–381.2]	<0.001 ^3^

Note: ^1^ independent samples *t*-test; ^2^ chi-square test or Fisher’s exact test; ^3^ Mann–Whitney U test. Abbreviations: BMI, body mass index; CRP, C-reactive protein; ESR, erythrocyte sedimentation rate; WBC, white blood cell count; M, male; F, female; SD, standard deviation.

**Table 2:** Clinical and immunological characteristics of patients with primary Sjögren’s disease (n = 34).

Variables	n (%)/Mean ± SD
ANA positivity	28 (82.4)
Anti-SSA positivity	18 (52.9)
Anti-SSB positivity	10 (29.4)
RF positivity	11 (32.4)
Low complement levels	2 (5.9)
Disease activity	
ESSDAI score	1.88 ± 0.95
ESSPRI score	6.13 ± 1.90
Patient global assessment (0–10)	5.82 ± 2.02
Physician global assessment (0–10)	4.29 ± 1.12

Note: Data are presented as mean ± standard deviation or number (percentage). Abbreviations: ANA: antinuclear antibody; SSA/SSB: anti-Ro/anti-La; RF: rheumatoid factor; ESSDAI: EULAR Sjögren’s Syndrome Disease Activity Index; ESSPRI: EULAR Sjögren’s Syndrome Patient-Reported Index; SD: standard deviation.

**Table 3:** Spearman correlation analysis between salivary galectin-9 levels and clinical, laboratory, and disease activity parameters in patients with primary Sjögren’s disease.

Variable	Spearman’s ρ	*p* Value	FDR-Adjusted *p* (BH)
Age (years)	0.148	0.402	0.669
Body mass index (kg/m^2^)	0.290	0.096	0.669
ESSDAI score	−0.342	0.048	0.669
ESSPRI score	−0.222	0.207	0.669
Erythrocyte sedimentation rate (mm/h)	−0.198	0.263	0.669
Patient global assessment (VAS)	0.085	0.633	0.779
Physician global assessment (VAS)	0.037	0.835	0.891
White blood cell count (×10^3^/µL)	−0.007	0.967	0.967
Neutrophil count (×10^3^/µL)	−0.131	0.460	0.669
Monocyte count (×10^3^/µL)	−0.071	0.689	0.787
Platelet count (×10^3^/µL)	−0.104	0.558	0.744
Lymphocyte count (×10^3^/µL)	0.148	0.405	0.669
Hemoglobin (g/dL)	−0.181	0.307	0.669
Red cell distribution width (%)	−0.149	0.402	0.669
Disease duration (years)	−0.142	0.425	0.669
C-reactive protein (mg/L)	0.160	0.367	0.669

Note: FDR (false discovery rate)-adjusted *p* values were calculated using the Benjamini–Hochberg (BH) method; an adjusted *p* value < 0.05 was considered statistically significant. Abbreviations: BMI, body mass index; ESSDAI, EULAR Sjögren’s Syndrome Disease Activity Index; ESSPRI, EULAR Sjögren’s Syndrome Patient-Reported Index; VAS, visual analogue scale. The nominal association observed with the ESSDAI did not retain statistical significance after false discovery rate correction.

## Data Availability

The data supporting this study are not publicly available due to ethical and privacy restrictions. Data are available from the corresponding authors upon reasonable request.

## Abbreviations

The following abbreviations are used in this manuscript:

pSD	Primary Sjögren’s disease
ESSDAI	EULAR Sjögren’s Syndrome Disease Activity Index
ESSPRI	EULAR Sjögren’s Syndrome Patient-Reported Index
ANA	Antinuclear antibody
RF	Rheumatoid factor
ESR	Erythrocyte sedimentation rate
CRP	C-reactive protein
Gal-9	Galectin-9
ROC	Receiver operating characteristic
AUC	Area under the curve
